# A case study in citizen environmental humanities: creating a participatory plant story website

**DOI:** 10.1007/s13412-021-00744-8

**Published:** 2022-02-10

**Authors:** Tina Gianquitto, Lauren LaFauci

**Affiliations:** 1 Department of Humanities, Arts, and Social Sciences, Colorado School of Mines, Golden, CO USA; 2 Department of Thematic Studies, Unit of Gender Studies, Linköping University, Linköping, Sweden

**Keywords:** Environmental humanities, Citizen humanities, Public participation, Environmental storytelling, Plant studies

## Abstract

Public engagement in crowd-sourced science projects such as iNaturalist or the Audubon Christmas Bird Count is a long-established practice within environmental studies and sciences. As a corollary to these “citizen science” efforts, “citizen humanities” engages public participation in humanities research and/or with humanities tools such as creative writing, photography, art-making, or conducting and recording interviews. In this essay, we outline our work creating a citizen environmental humanities website, *Herbaria 3.0*, including our motivations, process, and theoretical underpinnings. This project draws upon the critical understanding within environmental studies of the importance of narrative and storytelling for fostering a connection and commitment to environments and nonhuman beings. Situated within the field of environmental humanities, our website solicits, collects, and archives stories about the manifold relationships between plants and people, inviting visitors to read, share, or write their own story for digital publication. The kind of environmental storytelling that results, we argue, can (1) enrich our conceptualization of attachment to places, (2) expand our notion of what “counts” as an encounter with nature, and (3) help us recognize the agency of individual plants. We conclude that similar citizen humanities projects are crucial to the ongoing work of environmental humanities and environmental studies at large, for it is through such public engagement that we can meet the cultural challenges that seeded, and the societal problems occasioned by, ongoing climate change.

## Introduction

*Herbaria 3.0* (www.herbaria3.org) is a citizen environmental humanities website designed to encourage the sharing of plant stories from people around the globe. Situated within the growing field of environmental humanities, our website solicits, collects, and archives stories about the manifold relationships between plants and people, inviting visitors to read, share, or write their own story for digital publication. The project brings together emergent scholarly perspectives (citizen environmental humanities) and established approaches (environmental storytelling) of relevance to environmental studies. Public engagement in crowd-sourced science projects such as iNaturalist or the Audubon Christmas Bird Count is a long-established practice within environmental studies and sciences (Altrudi 2021; Nugent 2018). As a corollary to “citizen science,” “citizen humanities” encourages public participation in humanities research and/or with humanities tools such as photography, art-making, personal narrative interviews, and creative writing, to name just a few (Sze et. al. 2018, Tsing et al. 2017).

The *Herbaria 3.0* project draws on the critical understanding within environmental studies of the importance of narrative and storytelling for fostering a sense of place (Tooth and Renshaw 2009) and a connection and commitment to local and global environments and nonhuman beings (Lin and Li 2018; Satterfield and Slovic 2004; Basso 1996). Environmental narratives can convey complex environmental information (Anderson 2017; Lejano et al. 2013,), serve important memorial functions (Holmes and Goodall 2017), and preserve at-risk environmental/cultural histories (Arnold 2018). Further, the explosion of social media and other digital platforms in recent years has opened spaces for imaginative and creative engagement with both science and the natural world in digital spaces, which has in turn created an opening for citizen environmental humanities (Benmayor 2008).

*Herbaria 3.0* participates in growing efforts of environmental studies and environmental humanities scholars and educators to create spaces where people can share their personal experiences in nature. We argue that the kind of environmental storytelling that results from such engagement can (1) enrich our conceptualization of attachment to places, (2) expand our notion of what “counts” as an encounter with nature, and (3) help us recognize the agency of plant nature. We conclude that similar citizen humanities projects are crucial to the ongoing work of environmental humanities and environmental studies at large, for it is through such public engagement that we can meet the cultural challenges that seeded, and the societal crises that are occasioned by, ongoing climate change.

## Background: The *Herbaria 3.0* project origins

The *Herbaria 3.0* project began in 2016 as a group of colleagues from a range of disciplines, including literature, history of science, experiential learning pedagogy, geography, plant biology, and horticulture, converged to contemplate the role and meaning of plants in era of rapid climate change and species displacement. The initial international project team consisted of five members: Tina Gianquitto (USA), Lauren LaFauci (Sweden/USA), Dawn Sanders (Sweden/UK), Maura Flannery (USA), and Terry Hodge (USA).^1^ At the center of our starting discussions were a set of concerns: plants are everywhere in our world and in our lives, yet many people, according to plant biologists noting this problem over 25 years ago, fail to recognize or acknowledge their existence in the world at large (Wandersee and Schussler 2001). Globally, people spend millions on plant- and garden-related goods every year, and yet many remain insensitive to both the autonomous lives of plants and to the deeply textured natural-cultural history of plant-human interactions. The consequences of the perceptual bias against plants are tangible and profound (Sanders 2019). Plant conservation projects, for instance, receive less support than animal conservation projects (Belkin 2018; Balding and Williams 2016), and plants are often relegated to the “margin of the margin” of our intellectual and cultural histories (Marder 2013).

With these concerns in mind, and drawing upon the theoretical underpinnings supplied by the growing field of environmental humanities, the *Herbaria 3.0* team designed, constructed, and launched a digital platform for the collection and dissemination of plant stories, www.herbaria3.org. Initial funding for the project (ca. $40,000) was supplied in 2017 by the Seed Box: A Mistra-Formas Environmental Humanities Collaboratory centered at Linköping University, Sweden. Launched in 2018, the site draws upon a broad conception of “narrative” to encourage the sharing of all kinds of plant stories, including written stories, poems, photographs, and interviews and oral narratives; a linked Instagram account (@herbaria3.0) posts original images emphasizing human-plant interactions and invites other users to tag similar photos under the hashtag #alliseeisplants. As stories are submitted to the website, we curate them for the purpose of weaving each into a longer history of plant-human interactions by adding visuals, (e.g., digitized herbarium specimens, photographs, botanical drawings), historical/scientific correspondence, maps, and hyperlinks to other resources (such as open access databases and plant identification applications). Adding these layers to the website encourages visitors and participants to explore the history and science of plants and sparks recognition of human-plant relations over time.

The name of the site, *Herbaria 3.0*, emerged out of our shared interest in herbaria as historical records of human-plant interactions and out of our desire to echo the participatory, citizen-science activity of making herbaria themselves. Herbaria are collections of dried, pressed plant specimens, collected first by professionals and then by laypeople alike, for hundreds of years. As Barbara Thiers (2020) explains, the process of creating an herbarium specimen likely originated in Renaissance Italy through the efforts of physician and professor Luca Ghini, who sought a way to display key features of medicinal plants to his students even in winter, when access to fresh specimens was impossible. At that time, Ghini’s innovation marked a significant advance over even the most detailed botanical illustrations, as he placed a freshly gathered plant, including roots, stems, leaves, and flowers, in a naturalistic pose between sheets of paper, which were then pressed together to flatten the plant; once dried, the plant was glued onto a page in a blank book. Each herbarium page includes a label with important identifying information, including plant name (Latin and sometimes common names as well), distinctive characteristics, location and date of collection, and medicinal or other uses. These labels have become standardized on modern herbarium specimens (Thiers 2020).

This ancient technique for preserving specimens has changed little over time, and it remains the primary mode of preserving plants for scientific study. Herbaria were crucial for organizing and classifying the vast mass of plant life collected from large-scale colonizing expeditions of the seventeenth and eighteenth centuries (Batsaki et al. 2017, Schiebinger 2004). Later, with the boom of interest in botanic study in the eighteenth and nineteenth centuries, the making of individual herbaria for educational and recreational use became commonplace across Europe and the USA (Merrill 2008; Gianquitto 2007). In Sweden and Finland, making herbaria as part of school assignments remained a primary method of teaching local botany and natural history until fairly recently, and the process of making one’s herbarium was seen as both an important cultural heritage activity and a valuable practical exercise. Crucially, throughout their long history, herbaria have been shared social documents, circulating from collectors to scientists, and now, through digitization, to the public. These individual herbarium specimen sheets, as material artifacts of long plant-human relations, appear as the heading image of every story published on *Herbaria 3.0*: they are the “1.0” of our title.

Revolutions in archival organization and management occasioned by our current digital age mean that these material artifacts are available to more and more people via open access online databases, and the “2.0” of our project title recognizes this significant moment. As curators around the world make herbarium collections broadly accessible via digitization not only to scientists and historians, but also to artists and lay people, they bring together the material and historical with the digital and current, mixing media and opening new possibilities for research and creative interpretations. The process of digitization makes the herbarium specimen into what political ecologist Jane Bennett (2010) might call a vital object, one whose continued entanglements with humans assures that its meaning will always be subject to revision. Indeed, Bennett’s formulations of the radical entanglements of human and nonhuman, of vital materiality, and of distributed agency have been enormously influential to ESS scholars looking to tangle and untangle “the sticky web of connections” binding human and nonhuman activities (Bennett 2004). In the case of the herbarium specimen, as information is reviewed or newly determined, specimen labels are not removed; instead, new labels are added. The resultant multiplicity of labels on a single herbarium specimen thus itself becomes an archive of the shifting understandings of a particular plant specimen over time. We might also say that through the digitization process and subsequent participation of multiple contributors—through the “2.0” version of herbaria—the specimen becomes a kind of text, telling many stories of human-plant interactions across time and space.

Such stories can only be partially told through these specimen sheets, of course, which narrate or reveal a plant’s circulation in scientific knowledge cultures but which often isolate that plant from its historical and biocultural networks. Increasingly, researchers and others are reconnecting herbaria to their physical and historical contexts by, for instance, linking specimens to in situ plant and biocultural heritage inflection points (Cowell et al. 2020, Ryan 2015), rejoining specific specimens to botanical correspondence that accompanied them from global collection points (Clemit and Scott 2020; Gianquitto 2016), or, in institutional contexts, working to decolonize botanical gardens and natural history museums (Royal Botanic Gardens 2021; Caomhánach and Bell 2020). As a digital repository of personal environmental stories, *Herbaria 3.0* joins projects such as Center for Plants & Culture (plantsandculture.org), an online platform for educating visitors about the ways that plants shape and have shaped our culture over time, reflecting on economics, politics, law, and medicine, focusing on Black, Brown, and Indigenous voices (2021). These kinds of projects help highlight the erasures that happen in the colonization of plants and plant knowledge, specifically the erasures of traditional ecological knowledge (TEK) and of the histories of enslaved, indigenous, and colonized peoples, and of women, to name a few.

The herbarium sheet is thus a site of multi-layered richness, condensing into its two-dimensional forms both presence and absence in human-plant encounters—so much so that we used them as the organizing conceit of our website. In naming our project *Herbaria 3.0*, we aimed both to call attention to and participate in the long history of documenting plant-human relations: the “3.0” signifies our renewal of the archival, digital, and layered dimensions of thinking with herbaria over time. When visitors arrive at our website, they see a banner reflecting our project’s foundational belief, enticing individual contemplation: “Everyone has a story to tell about a plant: What’s yours?” They can then read a collection of stories written and submitted by ordinary people, browsing by date or by using tags and categories to find stories of interest to them. A prominent link to our submission page takes visitors to a simple form where they can create and submit their own plant story. Whether as readers, writers, or both, the visitors to our site become part of a “living archive” of plant-human relations. In this way, we share in the efforts of those who seek to redefine the archive in the Anthropocene from that of a privileged site of the “white Western academy” to a space defined “primarily in relationship to lived community practices and dynamic sites of cultural and creative production” (Almeida and Hoyer 2020, pp. 2, 3; Ryan 2015). We designed our digital platform as a similarly dynamic site, a kind of meeting space for the sharing of affective, material, and cultural responses to plants—and, potentially, as a driver of additional plant-human encounters in the offline, physical world. To date, we have received over 26,000 readers from over 130 countries.^2^

Just as one might consider an individual herbarium sheet as one layer in the historical record of human-plant encounters, and just as the large-scale digitization practices of these makes such records read-able new layers tracking such encounters over time, we imagined the stories of our site as adding a third layer to our natural-cultural^3^ understanding of plants. To call further attention to the project’s historical origins and inspirations, we included “Root Stories,” a section of the site that highlights historical narratives of encounters with specific plants. Because such a section could be unlimited in scale and scope, we tend to tie these stories whenever possible to the stories written in the here and now, the main focus of the site. For example, when we received a story about a Venus fly trap, Gianquitto wrote a root story post about American naturalist Mary Treat’s work with carnivorous plants in the nineteenth century (Gianquitto 2020). The vehicle of the particular plant thus serves as the connecting tie between the submitted story in the twenty-first century and the historical figure (and their narrative) many years prior. Along with the root stories, the historical herbarium specimen sheets accompanying each contemporary post reinforce the depth and longevity of human-plant relations across time and space. Both as an unfolding historical project and an ongoing cultural and environmental one, our project encourages visitors to recognize and respond to the subjectivities, curiosities, and presence of plants. With our site’s name acting as more, then, than simply an extended metaphor, *Herbaria 3.0* simultaneously facilitates the collection of primary sources (plant stories, plant photographs), creates a database for the documentation and sharing of these environmental narratives, and reframes plant archives as living, evolving, and creative cultural productions.

The site has been promoted widely to academic networks in arts, humanities, and sciences, on social media, and to public networks such as community gardens and gardeners, city and state botanic gardens, and conservation organizations. To aid those visitors to our site wishing to contribute but needing guidance to formulate a story “from scratch,” we created a “Resources” section where we share creative writing prompts and tips to help contributors craft their own stories. Finally, we also enabled the uploading of audio files to encourage interviewing of elders or anyone hesitant about writing a polished narrative for publication on the Internet. Like the “StoryCorps” project of NPR, we saw the audio option as a way to document experiences that might otherwise be lost to the ravages of time. For stories submitted as text, we established a practice of minimally editing authorial voices, allowing the original nuances of the storytellers to emerge without our interference.

We acknowledge that a digital resource for documenting and sharing plant stories could be seen as antithetical to larger goals within environmental studies to engage people with material, physical environments, especially considering the increasing prevalence of digital media over the last two decades—and exponentially so during the first 18 months of the Covid-19 pandemic. Some might argue further that collecting stories about real-world nature in the virtual space risks creating a barrier between actual experience of the natural world and the virtual expression of it. Digital, mediated experiences of nature are perhaps seen as less “real” and thus less valuable for the goals of creating awareness, knowledge, and empathy toward nonhuman nature. We acknowledge that such mediated interactions with nature might in fact reduce that experience of nature to a prescriptive, normalized one (Altrudi 2021). And of course, collectively, we are only just starting to realize the unanticipated and often negative consequences of employing digital technologies to satisfying our curiosity about nature: for example, conservationists are increasingly imploring people to stop geotagging social media images of remote locations and rare plants because of the resultant impacts of increased traffic to those areas (Holson 2018; McHugh 2016).

Nevertheless, digital media promise many benefits to environmental studies and sciences, including broader access to and easier dissemination of information, wider community reach, and public participation in research. These digital resources—including social media such as Instagram and TikTok, podcasts such as Ologies, and apps such as iNaturalist—are increasingly recognized as powerful tools in environmental work, particularly in increasing interest in the natural world and generating positive affective responses to it (Zhou and Li 2018). Within these platforms, specific users—like @alexisnikole (“The Black Forager”) on TikTok and @plants.and.culture, the associated account of the Center for Plants and Culture on Instagram—are exposing people to the interconnectedness of plant and human cultures over time, opening space in non-academic worlds for conversations previously held in academic departments about topics like the role of colonization in creating foodways.

Even as images of nature on social media today are carefully curated through the use of perspective, filters, and other visual editing tools, and even as we ourselves choose and edit images on *Herbaria 3.0* and our own Instagram feed, such images can still have positive impacts. Indeed, such editing is not new: from the beginnings of analog photography over 100 years ago, users have manipulated exposure times and cut negatives to capture ideal or idealized images of nature (Kozak 2019; LaFauci 2005). But these aestheticized or sanitized versions of the natural world are not necessarily detrimental to the aims of environmental studies and sciences. By exposing larger and more diverse audiences to the richness of environmental studies and sciences, such digital media encourage *increased* real-world engagement with nature. Similarly, the plant stories shared on our site and its associated Instagram account bind individual plants to individual lived experiences, creating powerful, regenerative connections between the human and nonhuman.

## Citizen environmental humanities: our approach and contribution

These design choices—to preserve authorial voices, to encourage different forms of media submission, and to create a digital resource in the first place—stem not only from our ambition to create a first-person living archive and database, but also from our desire to create a citizen environmental humanities project that increases the engagement of ordinary people with their surrounding environments. Such active participation in creating, collecting, and sharing knowledge about the natural world is part and parcel of our project’s situation in the field of environmental humanities. From the inception of this project in 2016, we envisioned the work within this field and strengthening one of its most crucial potential contributions, that of engaging citizens in environmental cultures.

While environmental humanities is a relatively recent area of academic inquiry, emerging only in the last decade or so, it grows out of advances in environmental studies at large (Heise 2017), particularly those emphases on the social imbrications of nature with culture. That is, just as environmental sciences expanded into environmental studies, environmental studies have now expanded to include environmental humanities, and with it, a broader array of humanistic subjects and approaches. The insights of environmental humanities enrich the field of environmental studies and sciences in their engagement with (for example), more-than-human and multispecies concerns, decolonial and indigenous perspectives, intersectional gender studies approaches, and artistic contributions to environmental awareness and engagement, among others.

Despite its relative youth as a field, environmental humanities research has erupted in the last decade, yielding at least two field-specific journals (*Environmental Humanities*, published by Duke University Press, in 2012; and *Resilience: A Journal of the Environmental Humanities*, published by University of Nebraska Press, in 2013); several graduate programs and international centers; and countless books, edited volumes, articles, conferences, and networks. This explosion of production has paradoxically made the field both more immediately understandable and more difficult to succinctly define. And this inability to map the field may itself be descriptive: there are many possible entrypoints to the environmental humanities—disciplinarily, of course, but also from within and beyond academia—and a tentacular abundance of approaches creating and developing it.

A foundational tenet of environmental humanities is that no single academic discipline is on its own enough to address or begin to solve our environmental crises. While the field collects under one umbrella a number of existing disciplinary subjects (literature, philosophy, history, political science, anthropology, sociology, geography, fine arts, gender studies) that deal with addressing environmental concepts, problems, or implications in qualitative ways (Rose et al. 2012), mere disciplinary “collection” cannot do or be enough to meet the environmental problems our societies face, enmeshed as they are in political, social, cultural, economic, historical, medical, and other systems. Our site recognizes this imbrication in two ways: theoretically, by bringing together project participants whose scholarship, teaching, and public practice are informed by a wide range of environmentally oriented research, and materially, by curating each story submission, adding relevant historical, political, social, cultural, and other contexts to the plant story.

A second, related tenet of environmental humanities, shared with environmental studies, is that nature and culture have always-already been enmeshed in one another, that these are not exclusive or objective concepts that can ever be understood without one another (Emmett and Nye 2017; Rose et al. 2012; Plumwood 2002). This point is perhaps the most significant for environmental humanities because the disciplines that make it up have for most of their disciplinary histories foregrounded human interests at the expense of nonhuman others. And of course, this point perhaps most significantly informs our site: the relationship between plants and people is not neutral and inert but provocative and active. That is, plants and people do not exist in disinterested relation to one another, but they actively create each other’s natural-cultural worlds.^4^ A third, related, tenet of environmental humanities (LaFauci and Åsberg 2020) that also underpins our site takes this point further: taking seriously the agency and value of nonhuman nature independent of human systems or even of human understandings. Environmental humanities work, alongside environmental studies, animal studies, and multispecies ethnography, has deepened and continues to deepen our conceptualizations of this “more-than-human” world, challenging as it does not simply the exceptionalism of humans but also the exceptionalism of “human” as a category independent of other organisms and beings.^5^ Here, the legacies of gender studies and specifically feminist science and technology studies are clearly evident, and many forebears of environmental humanities include feminist scholars such as Donna Haraway (2003), Val Plumwood (1993), Deborah Bird Rose (1992, 2004), Londa Schiebinger (1989, 1993), Anna Lowenhaupt Tsing (2015), and others. Indeed, shifting the focus away from humans and toward plants has been a key premise of the *Herbaria 3.0* project from the beginning.

We also wanted to shift the focus of “environmental knowledge” from the realm of expertise and science into the hands of ordinary people, empowering individuals to see themselves as creators and bearers of such knowledge. Recognizing that these environmental epistemologies are often highly localized, we share a commitment in our site to a fourth tenet of environmental humanities (LaFauci and Åsberg 2020): that all knowledge (and especially environmental knowledge) is situated. In its most self-evident sense, this situation is place-based, acknowledging that environmental knowledge changes with environments, with where one is situated in a place. But such situation also acknowledges the individual and collective experiences, and the intersectional factors that cross-cut these, that make knowledge subjectively determined. Importantly, acknowledging the situatedness of environmental knowledge necessitates that environmental humanities and studies scholars contend with coloniality and the manifold ways in which imperialism and genocide of indigenous peoples have disrupted traditional relations with(in) a given environment. In these ways, the field of environmental humanities could not have evolved without the influences of not only of feminist science and technology studies but also of indigenous scholars and elders, as well as of post- and decolonial scholars, often stemming from settler colonial nations such as Australia, the USA, and Canada. Recognizing that people differently experience, act in, and produce meaning about their experiences with plants, we designed our site to allow such subjectivities to emerge with as little editorial interference as possible, and with the underlying aim of uplifting, sharing, and archiving first-person experiences with the plant world.

Finally, our site shares a fifth goal of environmental humanities, a commitment to activism and public engagement, and of being relevant to non-academic audiences (Jørgensen and Jørgensen 2020; LaFauci and Åsberg 2020). Many scholars enact this commitment through participating in public protest, or by supplementing their academic work with writing for public audiences in, for example, op-eds or letters to the editor. Others target their academic “outputs” to audiences outside of academia, such as publishing them open access, creating free websites that “translate” the major conclusions or insights of book or article publications, giving free library talks, or forging collaborations with museums for exhibits and events. With the creation of the *Herbaria 3.0* website, we explicitly aimed to create a public-facing resource that had this important aspect of the field in mind: namely, that the site—informed by academic research in environmental humanities—would encourage active participation from readers and writers as both creators and consumers of a living archive of plant-human relations. We further hoped that the experiences of reading or writing for the site would in turn increase visitors’ capacity to notice, remember, and reconnect with plants in their everyday lives beyond the computer screen. Finally, we hypothesized that acknowledging plants and their ubiquitous presence in human worlds would help people learn more about plants’ place in their local ecosystems, thereby noticing other plants and slowly increasing their environmental appreciation and knowledge overall. While you can see elements of each aspect of environmental humanities reflected in our site’s creation and development, this fifth and final tenet perhaps best encapsulates our motivations and goals for *Herbaria 3.0:* to move from academic research to public engagement, to encourage those outside of university environments to see themselves as environmental thinkers and actors with important contributions to share with the world.

As academic disciplines, the environmental studies and sciences have long held promise for empowering individuals in this way and for joining the realm of research with policy and/or activism. It is understandable that humanities scholars in environmental subjects would also seek to make impacts here, for ethical reasons (the increasingly present and pervasive climate crises), professional ones (collaboration and trans-disciplinary work), and even for instrumental ones (such as funding opportunities). Since at least the 1960s, such public intellectual work bridging sciences and humanities has focused on bringing attention to environmental problems and policies. To take just one prominent example, Rachel Carson—trained as a scientist but writing for the general public with the skill of a poet and essayist—exposed connections between DDT and other chemicals with illness, arguably making irrevocable impact both on public awareness and on governmental policies. But despite the decades-long history of such public engagement in environmental problems, the particular promise of environmental humanities’ impact on environmental activism and policy has not been fully realized. Indeed, as Bergthaller et al. (2014) argue, “while environmental historians, environmental philosophers, and ecocritics (and those doing related work in neighboring disciplines) have enjoyed considerable success in *academic settings*, they have failed to reach a wider audience. When policy makers and mainstream media outlets seek expertise on the environmental crisis today, they seldom turn to environmental historians and philosophers, much less to ecocritics” (p. 262, our emphasis).

The gap between public environmental awareness or engagements and research in the library or lab might also stem from a deeper fracture between practice and theory in environmental work at large, given the rift between the urgency associated with environmental activism and the slower scholarship that characterizes most academic work (Posthumus et al. 2018). We need both the urgent and the deliberative, both activism and scholarship, of course, and it is our aim with the *Herbaria 3.0* website to combine the “slow scholarship” that we had undertaken prior to this project (in history of science, plant studies, and environmental humanities) with the faster, more responsive possibilities offered by digital humanities and environmental activism (Travis and Holm 2016). Our site invites creative, reflective, and personal environmental narratives from the public, producing and publishing them rapidly and accessibly in digital formats; in doing so, we join with other like-minded environmental humanities scholars to ask, “How can the intersection of technology, humanities, and ecological thinking yield new models of learning, research, and creative endeavor to model a dynamic knowledge ecosystem?” (Cenkl n.d.).

These intersections come together in the movement for “citizen environmental humanities.”^6^ In fact, these kinds of participatory efforts motivated the founding of the Seed Box Environmental Humanities Collaboratory that funded our pilot project: Founding Director Cecilia Åsberg envisioned the program as an example of feminist praxis (personal communication, 2016), where citizen humanities projects would stimulate the “cultivation of environmental imaginaries” and have the potential to encourage environmental engagements across diverse publics (Neimanis et al. 2015, p. 90). This new term—“citizen humanities”—accordingly emerged around the same time as that program (ca. 2015–2016) to define the efforts within humanities to move “across and between academia and other spheres of public engagements,” efforts that aimed to “reengage publics not only as consumers of environmental humanities research, but as its producers as well” (*Ibid*, pp. 88–89). This latter point, that the public must be involved not just in the consumption but also in the *production* of environmental knowledge, reveals the clear parallel between this newer realm and the more established one of “citizen science.” In both sets of projects, the public is deputized to participate directly in specialized research, albeit to varying degrees. In citizen environmental humanities, projects invite public production of humanities materials, such as environmental writing, photography or other art, sound recordings or interviews, and more.

In imagining our *Herbaria 3.0* website, we sought to highlight precisely these specific methods, practices, and tools of the humanities, providing a space to explore their contributions to environmental meaning-making, particularly around plant-human relations. While the public is often invited to participate in citizen environmental *science* projects—as in, for example, the Audubon Society’s international Christmas Bird Count, or in localized efforts like keeping watch over threatened nesting habitats of sea turtles—we realized that there were fewer opportunities to participate in environmental *humanities* research. Outside of museums, libraries, nature centers, or other public sites oriented around a particular (often local) environmental issue, there were few opportunities for members of the public to contribute to environmental humanities research and knowledge-making broadly. Our website thus aimed to be one node in such networks, one way for individuals to contribute, from the comfort of their own homes, to a shared archive of environmental stories, on the one hand, and to potential future academic environmental humanities research, on the other. By writing their own stories, or by documenting their connection to a plant photographically, contributors to our site (or users of our social media hashtag) would thus write their own plant story, see it published, and contribute to an environmental storytelling archive.

## Environmental storytelling as citizen humanities tool and result

The writing or recording of environmental stories is a way that humanities tools can be employed in the service of creating citizen-oriented projects, and, to that end, we see storytelling (and sharing, and archiving) as foundational to our project as both a method and a result. As we developed the *Herbaria 3.0* website, we began from a premise that environmental storytelling can foster knowledge- and meaning-making about plant-human interconnectedness in place and through time. Indeed, the original tagline of the site—“Where can a plant take you?”—invited such broad meditations on this interconnectedness, encouraging writers to journey through place, community, time, memory, and affect in their stories. At the same time, the phrase “a plant” was intentional: We hoped that by narrowing attention to an *individual* plant, as with an herbarium specimen page, specific dimensions of human and nonhuman relations would come into relief.^7^ A story about a Christmas cactus would highlight a difference set of relations than, say, a story about poison ivy. The stories collected on our site between spring 2018 and spring 2021 highlight plant-human relations and draw meaning from these relationships and experiences. From the body of work collected over these three years, we can draw several broad yet enmeshed conclusions about environmental storytelling in the citizen environmental humanities context:

1. “Place” in environmental storytelling has a particular richness, not limited to geographic location, that brings into focus the complex interrelations between and among human and nonhuman, rootedness and mobility, and memory, time, and affect.
2. The diverse and often domestic places of plant-human encounters—the “settings” of the plant stories—include yards, gardens, woods, fields, kitchens, porches, windowsills, mountains, and even grocery stores; these settings broaden our conceptualizations both of what counts as an encounter with nature and of who tells our nature stories or who gets to experience “nature.”
3. Directed focus on an individual plant or plant species highlights both that plant’s specific and unique qualities (scent, color, form) and its agentic role in the human-plant relationship.

### Expanding “place” beyond location: environmental storytelling and the promise of mobility

“Place-based narratives” have long been used in environmental studies contexts to foster critical engagement with local landscapes and ecologies (Gruenewald and Smith 2008) and to foster empathy with and an understanding of the nonhuman world. Initially, the Herbaria team anticipated that contributors to the site would engage with plants in the here-and-now of a particular place and time in the story-writer’s experience. What we found though, was that a plant in a story was more often dislocated from place, triggering instead memories of other landscapes and sets of relationships that existed in a writer’s past, and thus demonstrating the vivid entanglement of people and place in environmental memory. Places and plants together become “nodes in networks of meaning through which people link the meaningful places”—and people—“of their past and present lives” (Holmes and Goodall 2017, p. 5).

For instance, in “A Marigold of Mother” (2018), Giulia Lepori writes about marigolds that live simultaneously in her memories of childhood in Sardinia, Italy, and in the present space of her Australian deck. Lepori begins her story in memory: “As far as I recall, my mother has been planting tagetes [marigold] every Summer in Sardinia” (2018). The plant inspires a memory about the author’s mother sitting outside her home in the “narrow street” planting marigolds in pots. As Lepori learned about the use of marigolds in “natural agriculture” to “keep a healthy vegetable garden,” she connects the flowers to her mother’s “unconscious pioneering” of this traditional knowledge in her home village, and “WOW,” she writes: “Suddenly I saw them everywhere.” The connection is immediate and profound—but not specifically place-based.

Lepori, travelling around the world, not only sees the plants “everywhere,” but she also facilitates their movement herself: “It’s been three years since I have been planting marigolds of many species, sharing them with my mother from all over the places.” Perhaps the story proves Michael Pollan’s point in *The Botany of Desire* (2001): the marigold uses the author’s desire to connect with her mother, with “natural agriculture,” and with the role both have played in her life to expand their range. At the end of the story, Lepori indeed extends this range to the deck outside her new home in Australia. There she moves not just physical location but from past to present as well, in the process pointing out new experiences of the seasons in her new land: “It’s Winter here,” she writes, “but out in the deck the tagetes are about to bloom.” Lepori’s story reveals a pattern prevalent in many stories shared on our site: the physical location of the plant at the center of the story is generally less important to the storyteller—and to the storyteller’s main themes—than the memories, relationships, and personal reflections the plant inspires.

Lepori’s story also demonstrates that movements around the globe need not make environmental attachments more shallow: in fact, her moves actually deepen her relationship with the marigold plant and encourage her to seek more knowledge about its uses. This point is reinforced again and again in stories shared to our site, where people use plants as touchstones to discuss their deepened relationship with particular places or species, regardless of location or time spent in a single place. As we read the stories on our site, we realized that this trend ran counter to the tendency in much published nonfiction environmental writing that instead privileges long-term, stable, and unchanging relationships to one particular place or region. This privileging of one kind of relationship with place in turn threatens to marginalize the voices of those who do not have the same access to one place over a long period of time: those who must move to find or maintain employment, those who do not own land or property, those who are forced to migrate because of war, climate change, or threats of violence, to take just a few examples. Instead, the environmental storytelling that happens in places like our platform challenges ideas about time in relation to depth of experience in a place. Such mobility—among plants and people—could thus be seen not as a barrier to attachment to environments but instead as an opportunity to expand the locus of environmental care and concern beyond one’s “home” place toward broader, planetary thinking.

### Diversifying voices in environmental storytelling

As stories posted on the site drew our attention away from a focus on place to the network of relations that thinking about a plant inspired, we shifted the site’s tagline accordingly, to “Everyone has a story to tell about a plant: What’s yours?” The new focus concretely emphasizes our citizen humanities aims to address all who see our site and encourage them to participate in its collective meaning-making while still maintaining the focus on a singular plant experience. With this change, we also hope to counter a persistent belief, especially prevalent in the popular American imagination, that to “experience nature” requires removal from one’s everyday life to a separate “natural” or “wild” space where the more-than-human world is more prominent than the human one: think Chris McCandless in *Into the Wild* or Cheryl Strayed in *Wild*. Indeed, a persistent challenge we faced in getting members of the public to write a story for the website was a nagging concern that their experiences in the garden or with houseplants were not worthy of telling as a “nature story.”

This notion of what it means to be “in nature” is changing in both academic and in public spheres, with more attention being paid to the idea that nature has always-already been culturally constructed and imbricated in the political, social, and legal discourses of capitalism, colonialism, and slavery. This drive for inclusivity in natural spaces and expansion of what counts as a “nature experience” has been ongoing in the various disciplines of environmental studies and environmental activism for decades now. Influential scholarly challenges to the wilderness ideal persistent in both the popular American imagination and in its conservation/preservation practices include William Cronon’s “The Trouble with Wilderness, Or, Getting Back to the Wrong Nature” (1996), Ramachandra Guha and Juan Martínez Alier’s *Varieties of Environmentalism: Essays North and South* (1997), and Rob Nixon’s *Slow Violence and the Environmentalism of the Poor* (2011). The digital realm has increasingly hosted provocative challenges to defining what constitutes an experience of nature, including such grassroots groups as “Black AF in STEM” (2020), which hosts, for instance, a Black Birders Week (blackafinstem.com), “Black in Nature” (2021, www.blackinnature.com), and “Queer Nature” (2021, www.queernature.com), to name just a few. These efforts cross academic, public, digital, and natural spaces, although clearly more work is needed to make the cultural shifts necessary for all to experience equal access and safety in all spaces, natural and cultural.

For far too long, the voices of a vast majority of people whose experiences of nature do not fit into narrow archetypal categories like “wilderness” or “sublime” have been left out. We thus share with Gabriel Valle (2021) the conviction that sharing other kinds of encounters with nature opens space for “the voices, perspectives, realities, and ways of knowing that are often overlooked, silenced, or even erased by traditional environmentalism,” which has tended to privilege white, male voices (p. 131). This effect is magnified in our particular project, since the kinds of regular contact with plants that mark daily life would be otherwise elided from the category of “nature experience”: plants and people meet in the most ordinary of spaces, like gardens, streets, sidewalks, living rooms, and offices.

In her post “From Where I Sit,” author Anna Oberg (2018) explores precisely the tension between these different perceptions of nature. In the opening of the post, the author sits on a sofa in her living room, contemplating an abandoned plant on an end table, blooming in spite of her forgetting to water it. Her gaze travels from the plant to the curtain-covered window beyond it, from there to the deck with its patio furniture in disarray, and finally out to “where the sky meets the earth…a ridgeline sparsely slung with ponderosa pine.” “This,” she adds after a pause, “is my horizon.”

Here, Oberg contrasts the pull of experiencing an imagined space—“whatever roamed wild in the great blue yonder”—with the “the small, insignificances of domestic life” that define her actual circumstances. For Oberg, the “away” is where she “cultivate[s] charm, fascination,” where she understands her own “place in the wilderness of the world.” The comparative mundanity of home offered, she believed, no fodder for her creative life. But as her essay explores, the tension between the familiarity of home and the mystery of away is in fact more interrelated than she earlier perceived: the perspective of “out there *being* out there” becomes apparent only from “sitting in here” (our emphasis). That is, the pull to wander, and the corresponding environmental narrative of exploration and conquest, only gain meaning when there is “a point of return, a place to come back to” (2018). “There is no reason…to wander through the world,” she writes, “if nothing calls me back”—including the domestic insignificances she catalogues in the first part of her essay. Ultimately, she realizes “a portion of contentment”—and an ambivalent acceptance of an expanded locus of care—in “the act of finally rising, gliding across the room to pour leftover water on a dull plant.”

The prevalence of domestic settings like Oberg’s in the stories collected on our site was clear, even dominant. Such “domesticated environmental stories” represent the vast majority of our submissions. These kinds of stories privilege personal, highly specific encounters, often taking place within the walls of a home or in a public space like a street the storyteller walks daily. In domesticated environmental stories, writers extrapolate from this singular encounter to deeper meanings or larger reflections on relationships (whether between plants and people or between and among people in their lives). In “My Apartment’s First Guest,” for example, the plant fulfills a need for human connection, even community. It is in part a “sudden feeling of summer loneliness” that spurs author Taylor Nguyen (2020) to purchase the *Ficus lyrata* (Fiddle Leaf Fig) tree, implying that the plant was the companion who remedied that loneliness. In the close air of a small bedroom, human and plant support each other, whether through simple companionship or the symbiotic chemical exchange of oxygen and carbon dioxide: “we are able to synchronize our breathes in a way where he breathes in whenever I breathe out” (2020). When “other guests” arrive to Nguyen’s apartment, the tree is “the first thing they notice”; “he [the *Ficus* tree] greets them by glistening in the sunlight and reflecting the rays off of his leaves” (2020). Nguyen concludes that the *Ficus* tree is what transformed the relationship to the space itself: the tree “help[ed] me call my apartment home.”

As these two examples demonstrate, rather than existing “out there,” nature in the stories collected on *Herbaria 3.0* instead most often exists close to home. And as the authors on our site recognize the profundity of these nature encounters, they enact the kinds of cultural shifts necessary toward changing those constructions of nature that would situate it far away from such tame spaces. In these ways, the stories can be one small step toward the shifts environmental historian William Cronon called for over 20 years ago in his seminal article denouncing the separation of nature from culture, “The Trouble with Wilderness” (1996): “Idealizing a distant wilderness too often means not idealizing the environment in which we actually live, the landscape that for better or worse we call home. … In particular, we need to discover a common middle ground in which all of these things, from the city to the wilderness, can somehow be encompassed in the word ‘home’” (p. 21). The domesticated stories on our site play a role in creating this middle ground by linking “home” with profound experiences of natural connection; they thereby expand our cultural conceptions of what can be understood as an essential contact with nature, allowing for more varieties of nature experience to emerge. Over time, these stories could also change whose stories are told, read, and remembered in the first place.

### Witnessing plant agency

Locating stories in domestic spaces highlights rather than obscures the agency that plants possess in their relationships with humans. Perhaps this is because individual plants stand out in domestic spaces, where there tend to be fewer of them and their unique qualities are not lost in a green background. In the post above, Nguyen credits the “lively presence” of the houseplant in the corner of a bedroom for radiating “positive ambience,” for increasing “the flow of energy in the small space of my room,” for amplifying “a complete sense of well-being,” and finally, for helping “me call my apartment my home” (2020). As noted, plants in these stories are not inert bodies; rather, they are entangled in affectively charged interactions with human and other nonhuman actors. In “Message Sent in a Scent Envelope” (2019), author Dana Ecelberger describes an affective encounter with a plant that occurs at the threshold of the grocery store, turning a space for impulse purchases into an opportunity for reflection on rejuvenation and reciprocal care. The moment of entanglement is profoundly sensory—engaging first sight, then smell, and finally an imagined touch—and enables the writer to transcend what might otherwise be considered the commercialization or commodification of nature. Her encounter with a potted orchid instead inspires reflections on the plant’s own participation in the meeting: she gives it human qualities (“she” is “humble,” “generous,” and “subtle”), recognizing “her” individuality in comparison with the other “decadent” orchids; it is this difference that “*made me* put her in my shopping cart” (our emphasis). Ecelberger highlights the plant’s agency as directive over her actions: in the human telling, the plant orchestrates the encounter. Situated in a larger frame about missing past “plant friends” and their “messages,” the writer clearly views this orchid as a potential friend and/or communicative partner, one who called out to be purchased, and one who needs a bit of human tending—but who also can tend “her” human in the process.

The story’s title makes clear another prominent theme, that of the impact of scent, and scent’s role in plants’ communication with humans. Ecelberger notes that the plant releases scent as a way of nonverbally communicating with her: she smells the orchid in an otherwise mundane environment—the kitchen windowsill, a place of chores such as cooking and washing dishes. There, the plant repeatedly sends her “perfumed hugs,” which it releases seemingly in response to the author “thinking how pretty the orchid was.” That is, in the author’s understanding, the orchid was communicating with her, even reciprocating her affection. Current research on plant communication, namely the ability of plants to communicate with both other plants and non-plants through potent volatile organic compounds (Vivaldo et al. 2017), might indeed support the author’s sentiments here. Certainly, the orchid in Ecelberger’s story uses “creative, improvisational, and fleeting practices” (Hustak and Myers 2012) to involve itself in the author’s life: it “enveloped” her in “the most exquisite perfume,” causing her to “swoon[]” in a “bubble of fragrance.” When the scent recurs over a period of several days, Ecelberger becomes “convinced” that the orchid was “responding to [her] appreciation and admiration of it” (2019). Far from a mere commercial exchange in a grocery store, this story represents a profound encounter with the natural world, one that occasions reflection upon plant agency, communication, and care.

## Conclusion: Everyone *does* have a story to tell about a plant

Since its launch in 2018, the *Herbaria 3.0* site has met with a measure of success, with 117 submitted stories so far. A closer look at the submitted stories, however, shows that the site has resounded more with an academic audience than with a public one. Teachers at several institutions in the USA and Canada, for instance, have used the site for assignments involving environmental storytelling. When instructors share their assignments, we make them available on the site’s “Resource” pages. The Resources page also encourages readers to explore related sites, especially those that explore plant-human relations from different voices and creative perspectives, including art and photography. However, outreach beyond our current audiences is necessary if we are to meet our broad goal of contributing to environmental humanities’ efforts to engage diverse publics in our practices of research and artistic endeavors. We have identified one issue with connecting with this general audience: the name of the site and associated URL. Unless a person is already familiar with the term “herbaria,” it can be hard to understand—and to spell. So while “Herbaria 3.0” is a well-motivated and evocative name from an academic environmental humanities perspective, it does not evoke the same response from the general public. To address this problem, we plan to change the site URL to www.plantstories.org, and this new URL will send all visitors to the *Herbaria 3.0* page as it exists now.

But a more fundamental issue is at stake here as well. As noted above, we have encountered a general reluctance from people to recognize that the interactions they have with plants are worthy of telling a story about. Many people, when asked to contribute to the site, defer, demurring that they have nothing important to say. By encouraging people to contribute, by telling them that these small stories are worth sharing, we can stretch the potential for environmental storytelling beyond the three points we note above. Such storytelling, we believe, has the power to illuminate environmental changes happening in real time, and, on smaller scales, to make the invisible visible (Stoknes 2015), since it renders those changes more immediately legible to both storytellers and readers, than, say, the latest IPCC report. This process is vital if we are to generate the sustained commitments to our local environments that are necessary to meeting our shared, global problems. Can the abstract dilemmas of planetary climate change become more comprehendible when a person in Boston, in telling a story about a basil plant, meditates on how strange it is to see that plant emerging from a garden bed in January? We believe that by making visible and tangible the effects on plants resulting from environmental changes, our shared experiences and stories can move readers and storytellers toward the cultural shifts that are necessary for responding to current and impending climate crises.

As environmental studies scholars have long understood, such local, experiential dimensions of environmental storytelling highlight the power these stories have to reveal the depth of individual attachment to places—including, in this case, the plants in those places—as well as cultivate connections between and among individuals. The situatedness of environmental storytelling likewise can play a key role in diversifying our environmental narratives at large. In elevating voices of ordinary people, such stories can not only democratize environmental knowledge-making and enrich our environmental histories, but they can also—when collected en masse—reveal how these epistemologies and histories have always already been made by those on the margins of the official narratives. As Gabriel Valle points out, the process of collecting environmental stories makes apparent that “different people have different environmental histories, produce meaning about the environment through various but equally important stories, and perform or act differently in specific environmental contexts” (Valle 2021, p. 132).

Our interest in environmental storytelling as a tool and as an artistic product in its own right is thus entwined with our commitment to making *Herbaria 3.0* a citizen humanities project, one that positions the site as one node in larger environmental activist work. We believe that in focusing attention on singular plants, readers and writers generate other positive environmental outcomes, such as increased care for the more-than-human world, increased knowledge about local or distant environments, and perhaps even increased activism in their home communities. With or without realizing it, everyone does indeed have some kind of story to tell about a plant—the challenge of ours and other participatory environmental humanities projects is to create a space where those stories can be told so that this potential can be fully realized.

## Data Availability

Quotations from the environmental stories we cite in this article are freely available to the public via our website, www.herbaria3.org.

## Footnotes

1 Gianquitto and LaFauci possess expertise in historical and cultural studies of the social and political uses of plants. Sanders uses an art-based lens to research plant representation and learning in material contexts and has published as well on gardens as experiential learning spaces and science pedagogy. Flannery, a biologist, has written extensively on “plant blindness” and the botanical aesthetic, as well as on the ways that digitization has opened historic herbarium collections to wide audiences. Hodge, a biologist and horticulturist, brings practical experience with plants as well as scholarly and activist work in environmental justice.

2 Site statistics are current as of May 2021. From the outset, we also encouraged submission in the languages our team members could read: English, French, German, Italian, Spanish, and Swedish. We have received stories in English, French, Italian, and Swedish, from countries including the USA, Sweden, the U.K., Italy, Australia, New Zealand, and India.

3 Here, we are referencing Donna Haraway’s concept of *naturecultures* (2003), which argues for the inseparability of nature and culture, given that each is, at least in part, co-constructed by the other.

4 Studies of “plant agency,” or the capacity of plants to interact with and shape their environments, have grown significantly in recent years. Standard studies on plant agency include Marder (2013), Chamowitz (2012), Pollan (2001), and especially the works of Stefano Mancuso (Mancuso and Viola 2015, Mancuso 2018), Anthony Trewavas (2014), and Monica Gagliano (2013). Examples of studies of the co-creative dimensions of plant-human relations in both Western and non-Western and/or indigenous cultures include Arden and Miller (2020), Twelbeck (2020), Gibson and Baylee (2018), Nathen (2018), Attala (2017), Kawa (2016), Doody et al. (2014), and Ryan (2012).

5 Such challenges make the very moniker “humanities” a dubious one for many in the field, and concepts such as “posthumanities” have emerged in response (Åsberg 2001), marking an attempt both to shift away from anthropocentrism and to implicitly acknowledge the ways in which even the boundaries of our bodies are culturally, historically, socially, and politically determined.

6 In using the term “citizen,” we do not mean to exclude undocumented people, migrants, or others without ties to a nation-state. Instead, we view “citizen humanities” as a corollary term to “citizen science,” a widely understood term for the democratizing of and public participation in scientific knowledge production. Within citizen science, this usage is subject to increasing discussion and deliberation, and some are moving to change the term to, for example, “Community and Citizen Science, “Civic Science,” “Community-based Participatory Research” (CBPR) or “Public Participation in Scientific Research” (PPSR) (Eitzel et al. 2017. p. 7; U.S. Forest Service, n.d.). However, because this debate is not the focus of our argument here, we will simply note our cautious use of the term “citizen environmental humanities,” and, following Eitzel et al. (2017), note that we use “citizen” throughout to indicate not a legal category but a “member of a broadly construed community” (p. 6).

7 *Herbaria 3.0* Co-PI Dawn Sanders originated this tagline and focus on a single plant (“Where can a plant take you?”), an outgrowth of her project “Beyond Plant Blindness: Seeing the Importance of Plants for a Sustainable World” (funded by Vetenskapsrådet / The Swedish Research Council, 2013–2014).
